# Efficacy of 2-(1-hexyloxyethyl)-2-devinyl pyropheophorbide-a in photodynamic therapy of human esophageal squamous cancer cells

**DOI:** 10.3892/ol.2013.1493

**Published:** 2013-07-25

**Authors:** DENGPAN WU, ZHEN LIU, YANNI FU, YUAN ZHANG, NAN TANG, QIN WANG, LIANG TAO

**Affiliations:** 1 Department of Pharmacology, Zhongshan School of Medicine, Sun Yat-Sen University, Guangdong, Guangzhou 510080, P.R. China; 2 Department of Anesthesiology, Sun Yat-Sen Memorial Hospital, Sun Ya-Sen University, Guangdong, Guangzhou 510120, P.R. China

**Keywords:** 2-(1-hexyloxyethyl)-2-devinyl pyropheophorbide-a photodynamic therapy, human esophageal squamous cell cancer

## Abstract

The present study investigated the effects of 2-(1-hexyloxyethyl)-2-devinylpyro pheophorbide-a (HPPH)-mediated photodynamic therapy (PDT) on *in vitro* cell survival and *in vivo* tumor growth derived from human esophageal squamous cancer cells (Eca109). A cell counting kit 8 (CCK8) assay was used to assess the phototoxicity of HPPH-mediated PDT in cultured Eca109 cells. The inhibition of tumor growth was determined by the changes in the relative tumor volume (RTV) and tumor weight. The results revealed that HPPH, in the range of 0.005–1 μg/ml, exhibited no cytotoxicity in the Eca109 cells without light exposure and that the *in vitro* efficiency of HPPH-mediated PDT was higher compared with that of Photofrin^®^-mediated PDT. The *in vivo* results indicated that graded doses of HPPH-mediated PDT significantly inhibited the xenograft tumor growth derived from the Eca109 cells in a dose-dependent manner. The inhibition efficacy of 0.6 and 1.0 mg/kg HPPH-mediated PDT was similar to that of 10 mg/kg Photofrin-mediated PDT. Furthermore, HPPH possessed a lower toxicity than Photofrin at the dose that achieved the same efficacy in mice bearing Eca109 subcutaneous tumors. The histopathological findings indicated that the tumor tissues in the photosensitizer (PS)-treated mice demonstrated varying degrees of necrosis. HPPH and Photofrin exhibited vascular cytotoxicity on the treated tumors. In conclusion, the present study demonstrated that the phototoxicity of HPPH-mediated PDT is higher than that of Photofrin-mediated PDT of the same dose. HPPH possessed lower toxicity than Photofrin at the dose that achieved the same efficacy. Therefore, HPPH may be a promising agent for treating human esophageal squamous cell cancer (ESCC).

## Introduction

Human esophageal squamous cell cancer (ESCC) is one of the most prevalent cancers in the world and ~250,000 ESCC cases are diagnosed each year in China, accounting for half of the world’s cases (1). However, the optimal treatment for squamous cell carcinoma of the esophagus remains unresolved. For the majority of ESCC cases, surgery remains the first choice of therapy (2,3). However, the results of surgery at the five-year follow-up period remain poor, regardless of the type of surgical intervention (4). Furthermore, a significant proportion of patients are not eligible for surgery due to a delay in the diagnosis of the tumor. Others may have an early-stage cancer but are considered unsuitable for surgery due to comorbid disease. Chemotherapy and external radiotherapy are suitable for only a small proportion of patients. Consequently, photodynamic therapy (PDT), a technique consisting of an application of a light source following the prior administration of a photosensitizing drug, which is able to induce necrosis of the targeted tissue (5,6), may have a role in the management of patients with esophageal cancer who pose a high surgical risk (7). Photofrin^®^-mediated PDT was first approved by the FDA in 1995 for the palliation of symptoms and the reduction of obstruction in patients with completely- or partially-obstructing esophageal cancer. However, PDT is also associated with prolonged and occasionally severe cutaneous phototoxicity in patients (8). This limitation has been the major impetus behind the synthesis of new sensitizers with a higher efficacy and lower phototoxicity.

**Figure 1:**
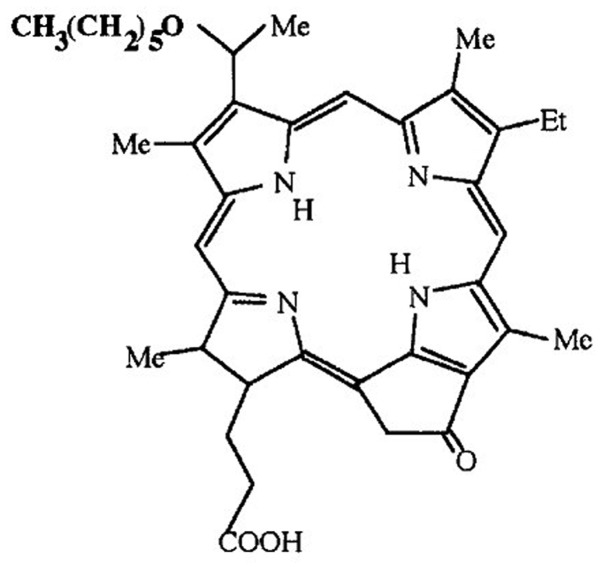
Chemical structure of 2-(1-hexyloxyethyl)-2-devinyl pyropheophorbide-a (HPPH).

As a second-generation chlorin-based compound, 2-(1-hexyloxyethyl)-2-devinyl pyropheophorbide-a (HPPH; Fig. 1), has shown favorable photophysical and pharmacokinetic properties in preclinical studies (9,10). HPPH has been reported to be an extremely hydrophobic compound that is the most effective photosensitizer (PS) against murine tumors amongst a series of homologues with various numbers of methylene groups on the ether function (10). The compound strongly absorbs light at 665 nm. Therefore, light penetration into the tumor tissue is increased compared with Photofrin (11). In addition, HPPH may exhibit lower skin phototoxicity since it has been shown to be rapidly cleared from the skin (12). Clinical phase I and II studies of HPPH that were conducted in patients with Barrett’s esophagus and obstructive esophageal carcinoma have indicated excellent response rates (13). However, to the best of our knowledge, no prior publication has investigated the effects of HPPH-mediated PDT on human ESCC.

The present study aimed to investigate the efficacy of HPPH in PDT of human esophageal squamous cancer cells (Eca109) *in vivo* and *in vitro*, therefore providing additional evidence for the study of HPPH-mediated PDT on human ESCC.

## Materials and methods

### PSs

Photofrin (sterile freeze-dried powder, 75 mg/vial) was purchased from the Axcan Pharma Company (Birmingham, AL, USA) and freshly prepared in 5% dextrose solution in the dark prior to use. HPPH freeze-dried powder and HPPH vehicle, provided by Zhejiang Hisun Pharmaceutical Co., Ltd. (Zhejiang, China) were freshly diluted using sterile 0.9% normal saline (NS).

### In vitro photosensitivity activity

The human Eca109 cell line was purchased from the Shanghai Institute for Biological Sciences (Chinese Academy of Sciences, Shanghai, China). The cells were maintained in RPMI-1640 medium (Gibco, Carlsbad, CA, USA) containing 10% FBS, 2.5 mg/ml glucose, 0.11 mg/ml sodium pyruvate and 1% penicillin-streptomycin, and cultured at 37°C in a humidified 5% CO_2_ incubator (Thermoscientific, Waltham, MA, USA). The cells in the log phase of growth at 70–90% confluency were inoculated in a 96-well microplate. Following overnight incubation, the cells were divided into a light exposure group and a no light exposure group. Each group contained cells that were incubated with HPPH vehicle or variable concentrations of HPPH or Photofrin. All groups were incubated at 37°C for 24 h without exposure to any light. For the exposure to light, the initial incubation media was replaced with drug-free complete media prior to the light treatment and then illuminated with light from an argon-pumped dye laser set at 665 nm for HPPH or 630 nm for Photofrin at a fluence rate of 20 mW/cm^2^ for 2 J/cm^2^ (2.5 cm-diameter illumination). Following PDT, the cells were cultured for a further 48 h at 37°C in the dark. For the cells that were not exposed to light, following a replacement of the initial incubation media with drug-free complete media, the cells were cultured for a further 48 h at 37°C in the dark. Following the 48-h incubation period, a cell counting kit 8 (CCK8) assay was used to assess the phototoxicity of HPPH to the cells. Briefly, 10 μl CCK8 solution (Dojindo Laboratories, Kumamoto, Japan) was added to each well and the 96-well plate was continuously incubated at 37°C for 1 h. The OD value for each well was read at a 450 nm wavelength to determine the cell survival rate on a microplate reader (Epoch; Biotek, Winooski, VT, USA). The assay was repeated three times. An IC_50_ value was calculated using Origin 7.5 software (OriginLab, Northampton, MA, USA).

### In vivo photosensitizing activity

The six to eight-week-old BALB/c-nude mice were provided by Guangdong Laboratory Animal Centre (Guangdong, China) and housed under specific pathogen-free conditions throughout the study, at 22–24°C in 50% humidity. This study was approved by the ethics committee of Sun Yat-Sen University (Guangzhou, China). The mice were inoculated subcutaneously under the right shoulder with 5×10^6^ Eca109 cells in 200 μl serum-free medium. The *in vivo* antitumor photosensitizing efficacy of HPPH-mediated PDT was evaluated when the volumes of the tumors ranged between 100 and 300 mm^3^. The mice were injected intravenously with 0.9% sterile NS, which was used as a negative control, HPPH vehicle (1 mg/kg of body weight; control), varying doses of HPPH (0.15, 0.3, 0.6 and 1 mg/kg of body weight) or Photofrin (10 mg/kg of body weight; positive control). At 24 h post-injection and without exposure to light, the mice were irradiated with a laser light from an argon-pumped dye laser set at 665 nm for HPPH or 630 nm for Photofrin. The treatment parameters consisted of a light spot of 1.6 cm diameter and a total light dose of 135 J/cm^2^ delivered at a fluence rate of 75 mW/cm^2^(14,15). Following PDT, the tumor dimensions were measured using calipers every four days. The tumor volume (TV) was calculated with the following formula: TV = (L × W^2^) × 0.5, where L is the longest axis of the tumor and W is the axis that is perpendicular to L. The relative tumor volume (RTV) of each tumor was defined as the ratio of the volume at a given time to the volume at the start of treatment (16). The mean RTV was calculated for each treatment group. The antitumor activity was determined by calculating the tumor growth inhibition (TGI) value using the following equation (16,17): TGI (%) = T/C × 100, where T is the mean RTV of the treated tumors at the end of the experiment (three weeks) and C is the mean RTV of the control group. The xenograft tumors were excised and weighed subsequent to the mice being humanely sacrificed at the end of the experiment. The tumors were weighed and the weight inhibition value was calculated using the following equation: Tumor weight inhibition (%) = 1 − [mean tumor weight (experiment groups)/mean tumor weight (HPPH vehicle group)] × 100.

The present study was performed according to the document Guidance Suggestions for Caring for Laboratory Animals produced by the Ministry of Science and Technology in 2006.

### Toxicity following HPPH-mediated PDT

To evaluate the toxicity following HPPH-mediated PDT, the body weights of the mice in each group were recorded every four days subsequent to the treatments. Furthermore, the mortality rate of the mice in each group was recorded daily over the three-week treatment period and the percentage of lethality was defined as the ratio of the total amount of dead animals at the end of the experiment to the total amount of animals at the start of the treatment.

### Histology following HPPH-mediated PDT

To gauge the pathological effects of HPPH-mediated PDT, several animals were selected at random from each group and sacrificed at the end of the experiment. The tumors were excised and fixed in formaldehyde-mixing fixative for 24 h, then rehydrated and embedded in paraffin. Representative sections of tumor were stained using hematoxylin-eosin (HE). The results were observed under ×40 or ×400 magnification using a light microscope.

### Statistical analysis

The experimental data in each group are presented as the mean ± SD. An analysis of the variance between groups was performed with SPSS software (SPSS, Inc., Chicago, IL, USA) for windows 11.5 using Student’s t-test or a one-way ANOVA. P<0.05 was considered to indicate a statistically significant difference.

## Results

### Effects of the incubation time of drugs prior to and following light exposure on the phototoxicity of HPPH-mediated PDT

**Figure 2:**
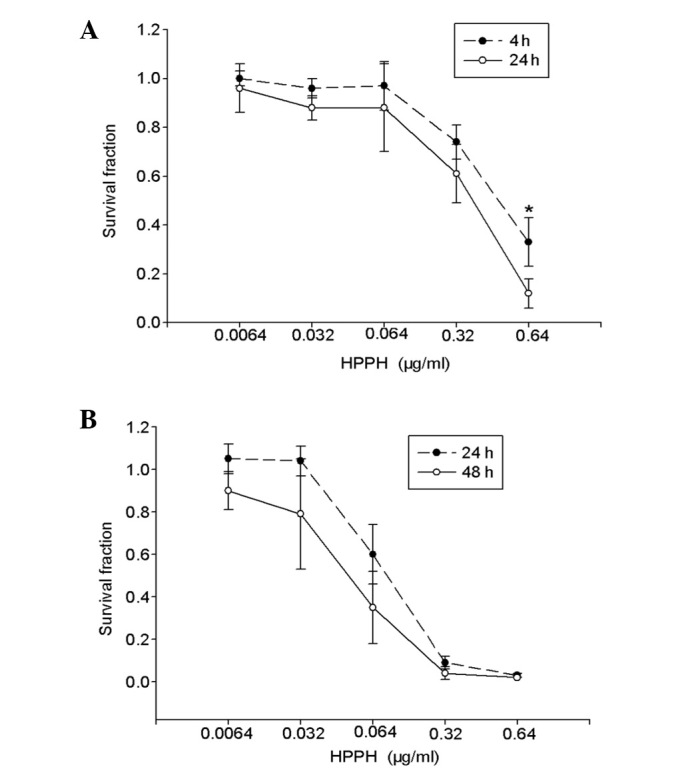
(A) Effects of the incubation time of drugs prior to light exposure. (B) Effect of times following light exposure on the phototoxicity of HPPH-mediated PDT. Data are presented as the mean ± SD (n=3). Statistically significant differences were calculated by Student’s t-test using SPSS 11.5 software and are indicated by ^*^P<0.01. HPPH, 2-(1-hexyloxyethyl)-2-devinyl pyropheophorbide-a; PDT, photodynamic therapy.

An intracellular PS has been considered as a factor to determine the efficacy of PDT. The incubation time of drugs usually affects the intracellular uptake of the PS. Accordingly, in order to evaluate the effect of the incubation time on the phototoxicity of HPPH, the Eca109 cells were incubated with HPPH for 4 h and 24 h, respectively, prior to the light exposure. Subsequent to being exposed to light at a dose of 2 J/cm^2^ (2.5 cm-diameter illumination), the cells were cultured in the dark for a further 24 h. The cell viability was then determined using a CCK8 assay. As shown in Fig. 2A, the survival fraction of HPPH following a 24-h incubation period was higher than that of a 4-h incubation.

### Incubation time following light exposure may play a role in the phototoxicity of PS

To assess the effect of the incubation time following light exposure on the phototoxicity of HPPH, the Eca109 cells were incubated with various doses of HPPH for 24 h. Following light exposure, the cells were cultured for a further 24 h or 48 h in the dark. As shown in Fig. 2B, there was no significant difference in the phototoxicity of HPPH between the 24-h and 48-h incubation periods following light exposure.

### Effect of the dose of the exposed light on the phototoxicity of HPPH-mediated PDT

**Figure 3:**
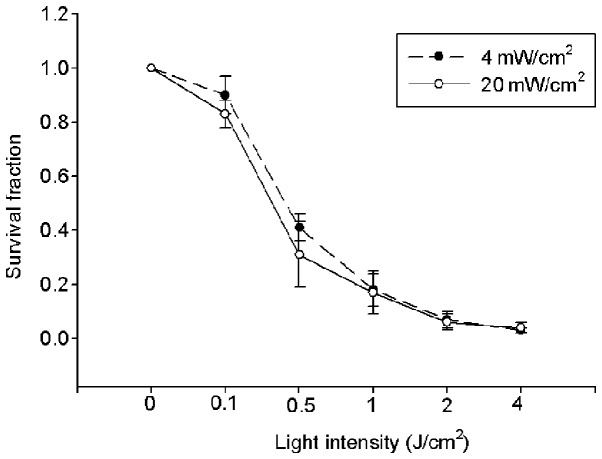
Effect of the dose of the light exposure on the phototoxicity of HPPH-mediated PDT. Data are presented as the mean ± SD (n=3). HPPH, 2-(1-hexyloxyethyl)-2-devinyl pyropheophorbide-a; PDT, photodynamic therapy.

The dose of light energy and the rate of energy delivery have been recognized as pivotal factors to determine the biological consequences of PDT. To investigate the effect of light intensity on the phototoxicity of HPPH *in vitro*, the cells were illuminated with various light doses (0.1 J/cm^2^–4 J/cm^2^) at a fluence rate of 4 mW/cm^2^ or 20 mW/cm^2^ following a 24-h incubation period with 0.192 μg/ml HPPH in the dark. Following illumination, the cells were cultured for a further 48 h in the dark and the cell viability was assessed using a CCK8 assay. As shown in Fig. 3, the inhibition rate of HPPH-mediated PDT was increased in a light dose-dependent manner. However, there was no significant difference between the two dose rates.

### In vitro photosensitivity activity

**Figure 4:**
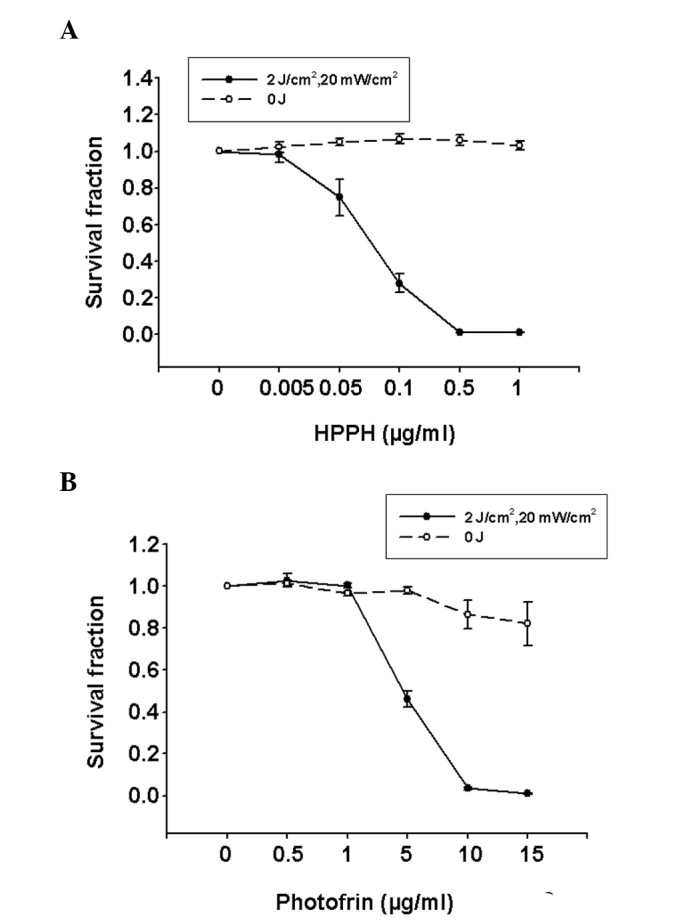
Efficacy of HPPH and Photofrin^®^-mediated PDT in Eca109 cells. Data are presented as the mean ± SD (n=3). HPPH, 2-(1-hexyloxyethyl)-2-devinyl pyropheophorbide-a; PDT, photodynamic therapy.

In order to evaluate the efficacy of HPPH- and Photofrin-mediated PDT in human ESCC cells, the Eca109 cells were incubated with various concentrations of HPPH (0.005–1 μg/ml) or Photofrin (0.5–15 μg/ml) for 24 h in the dark prior to being exposed to a laser light at 665 nm or 630 nm (20 mW/cm^2^, 2 J/cm^2^). The cells were then cultured for 48 h and the *in vitro* photosensitizing efficacy was determined using a CCK8 assay. As shown in Fig. 4, the cells with no light exposure displayed no significant toxicity with up to 1 μg/ml HPPH and 15 μg/ml Photofrin, which is similar to the results previously demonstrated in other kinds of ESCC cells (26). However, subsequent to being activated by light, HPPH and Photofrin were able to significantly inhibit cell survival in a concentration-dependent manner. The IC_50_ of HPPH-mediated PDT was 0.07 μg/ml, whereas the IC_50_ of Photofrin was 3.94 μg/ml. Compared with Photofrin, HPPH had a higher efficacy in the PDT of the Eca109 cells *in vitro* (Fig. 4).

### In vivo photosensitizing activity

The *in vivo* photosensitizing efficacy was determined in the BALB/c-nude mice that were transplanted with Eca109 tumors. When the tumors reached 100–300 mm^3^, the mice were injected with HPPH at various drug doses and exposed to light (665 nm, 135 J/cm^2^, 75 mW/cm^2^) at 24 h post-injection. Photofrin (10 mg/kg) was used as a positive control and mice were exposed to light (630 nm, 135 J/cm^2^, 75 mW/cm^2^) at 24 h post-injection. The tumor growth was monitored by measuring the TV every four days for three weeks and the mean RTV was calculated for each treatment group. As shown in Table I, HPPH was able to inhibit the tumor growth in a dose-dependent manner. Doses of 0.6 mg/kg and 1 mg/kg HPPH-mediated PDT were highly effective in controlling the tumor growth from day 5, which was similar to the Photofrin-PDT dose of 10 mg/kg.

The PDT efficacy was estimated by the TGI at three weeks post-treatment, which was calculated by the formula described in the methodology section. The National Cancer Institute standard for the minimal level of antitumor activity (TGI ≤42%) was adopted. As shown by Table II, at day 21, the TGI values of the NS and 0.15 mg/kg HPPH groups were 92.91 and 74.49% respectively, which was higher than the 42% minimal level. The TGI values of the mice that were treated with HPPH (0.3 mg/kg, 0.6mg/kg or 1mg/kg) and Photofrin (10 mg/kg) were 30.24, 1.18, 1.35 and 1.18% respectively, which were also lower than the 42% minimal level. This data demonstrated that a HPPH dose of 0.6–1 mg/kg has a comparable PDT efficacy *in vivo* with that of 10 mg/kg Photofrin, which is currently used in clinics.

**Figure 5:**
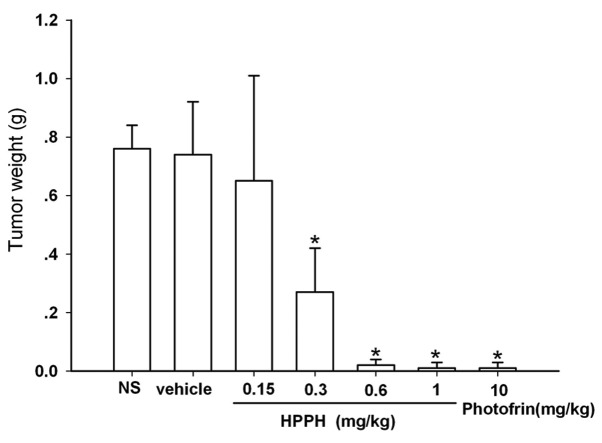
The tumor weights of mice following HPPH- and Photofrin^®^-mediated PDT. ^*^P<0.05, vs. the HPPH vehicle group. HPPH, 2-(1-hexyloxyethyl)-2-devinyl pyropheophorbide-a; PDT, photodynamic therapy; NS, normal saline.

At the end of the treatment period, the tumor weights of the mice treated with PDT and HPPH doses ranging from 0.3–1 mg/kg or 10 mg/kg Photofrin had remarkably decreased compared with those of the vehicle group (Fig. 5). The inhibition rates of the tumor weights following 0.6 mg/kg and 1 mg/kg HPPH- and 10 mg/kg Photofrin-mediated PDT reached 97.3, 98.65 and 98.65% respectively (Table III). However, the tumor weights in the mice following PDT with NS or 0.15 mg/kg HPPH did not show significant differences compared with those of the vehicle group (Fig. 5).

### Effects of PDT with various doses of HPPH on mouse lethality and body weight

The mean body weights of the mice decreased rapidly following the administration of HPPH- and Photofrin-mediated PDT (Table IV). In addition, as shown in Table V, the percentage of lethality induced by 0.15, 0.3 and 0.6 mg/kg HPPH-mediated PDT was 37.5%. Notably, high lethality was observed during the experimental period in mice following 1 mg/kg HPPH-mediated PDT (62.5%) and 10 mg/kg Photofrin-PDT (50%; Table V).

### Gross and histological observation of tumors following PDT

**Figure 6:**
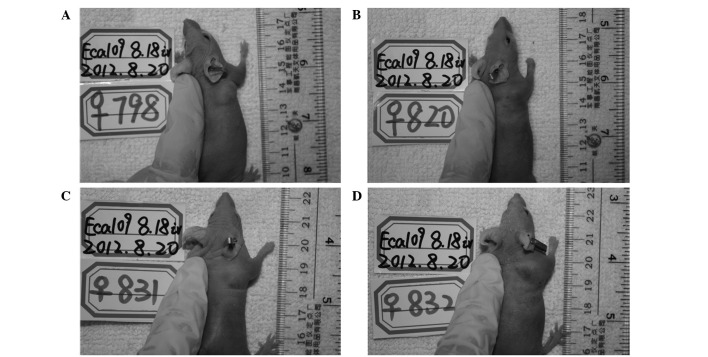
Edema surrounding the tumors at day 1 post-PDT. Mice bearing representative tumors were randomly selected from each group. (A) no edema, (B) slight edema, (C) moderate edema and (D) severe edema. PDT, photodynamic therapy.

PDT was performed at 24 h post-PS administration and the reactions of the tumor and mice following PDT were recorded. No direct injuries, including capillary rupture or hemorrhagic effusion, were observed at the surface of the tumor nodules or in the vicinity over the three-week treatment period. In addition, edema surrounded the tumors in the mice that were injected with PS, including 0.3–1 mg/kg HPPH and 10 mg/kg Photofrin, at day 1 following PDT. However, there was no edema in the mice subsequent to PDT with NS, vehicle and 0.15 mg/kg HPPH. In the mice with subcutaneous edema, the degree was different. As shown in Table VI and Fig. 6, half of the mice exhibited severe edema following PDT with 1 mg/kg HPPH or 10 mg/kg Photofrin, while in the groups with PDT and 0.3 or 0.6 mg/kg HPPH, mice with slight edema accounted for 50%.

**Figure 7:**
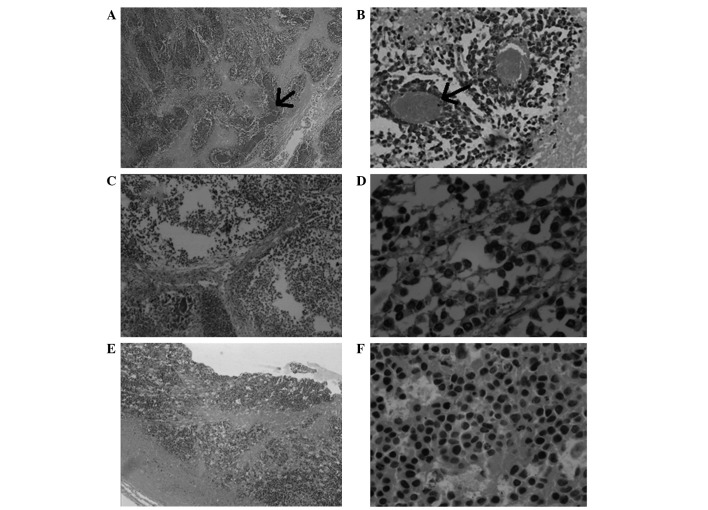
Micrographs of 10-mm slices of xenografted tumors derived from Eca109 cells that were stained with hematoxylin-eosin (HE) and observed under (A, C and E) ×40 or (B, D and F) ×400 magnification using a light microscope. (A and B) PDT with HPPH vehicle or NS. (C and D) PDT with 0.15 or 0.3 mg/kg HPPH. (E and F) PDT with 0.6 or 1.0 mg/kg HPPH or 10 mg/kg Photofrin^®^. Samples were selected at random from each group. Black arrows indicate tumor vessels. HPPH, 2-(1-hexyloxyethyl)-2-devinyl pyropheophorbide-a; PDT, photodynamic therapy; NS, normal saline.

To evaluate the pathological effects of HPPH-mediated PDT, several animals were selected at random from each group and sacrificed at the end of the experiment. The tumor tissue was removed from the mice and the tissue damage induced by PDT was assessed histologically. The histopathological findings indicated that the tumor tissues in the PS-treated mice demonstrated varying degrees of necrosis, while the untreated tumor tissues were filled with dense tumor cells, in which a basophilic cytoplasm was observed (Fig. 7A and B). Furthermore, as illustrated in Fig. 7C and D, less basophilic cytoplasm was observed in the tumors that were treated with 0.15 or 0.3 mg/kg HPPH. A low density of tumor cells and eosinophilic cytoplasm was observed in the tumors following PDT with 0.6 or 1 mg/kg HPPH or 10 mg/kg Photofrin. The histopathological results also demonstrated that tumor vessels (arrows, Fig. 7A and B) were observed in the control and negative control groups (mice injected with vehicle or NS). Tumor vessels were not observed in the PS-treated tumor tissues (treated with 0.6 or 1 mg/kg HPPH or 10 mg/kg Photofrin; Fig. 7E and F).

## Discussion

Surgery remains the first choice of therapy for patients with ESCC, even though the five-year follow-up prognosis remains poor. HPPH treatment, which has an increased penetration and lower skin phototoxicity compared with Photofrin (11,12), has been conducted in patients with Barrett’s esophagus and obstructive esophageal carcinoma and has indicated excellent response rates (13). However, the effect of HPPH-mediated PDT on ESCC remains unknown. The present study aimed to investigate the effects of HPPH-mediated PDT on the survival of Eca109 cells and the growth of xenograft tumors derived from Eca109 cells in mice.

Photofrin cellular concentrations have been reported to decrease exponentially in certain cell lines on a time-dependent basis (18), which may result in a decreased cytotoxicity of PS. However, in another study, following a 24-h incubation period, the intracellular concentrations of HPPH were higher than after 4 h of incubation in colon26 cells (19). The present study revealed that the survival fraction of HPPH-treated cells following 24 h of incubation was higher than for 4 h of incubation (Fig. 2A), which may be attributed to the increased uptake of HPPH.

Cells may be able to initiate a rescue response and/or undergo cell death in an apoptotic or necrotic fashion following photodynamic damage (5,20). The present study showed that there was no significant difference in the phototoxicity of HPPH between the 24-h and 48-h incubation periods following light exposure (Fig. 2B), which may be due to the cells initiating a rescue response, including (de)phosphorylation, changes in second messengers, such as calcium and cAMP, and the activation of proteins by proteases (21).

Several studies have demonstrated that high-fluence rate PDT may lead to treatment-limiting deficits in the available oxygen if the high rate of ^1^O_2_ generation outpaces the resupply of O_2_(22,23). In the present study, no significant differences were observed between the cells that were exposed to light at doses of 4 mW/cm^2^ or 20 mW/cm^2^ (Fig. 3), indicating that HPPH-mediated PDT at a fluence rate of 20 mW/cm^2^ may result in treatment-limiting deficits in Eca109 cells.

Numerous lines of evidence have indicated that HPPH-mediated PDT has shown significant cytotoxicity *in vitro* and antitumor efficacy in a number of tumor xenograft models (24–27). Furthermore, HPPH has been demonstrated to possess greater cytotoxicity per drug dose than Photofrin (24). In the present study, HPPH and Photofrin-mediated PDT were observed to exhibit a high cytotoxicity *in vitro* and antitumor efficacy *in vivo* in a concentration-dependent manner (Tables I–III; Fig. 4). In addition, HPPH-mediated PDT had a higher efficacy than Photofrin-mediated PDT in the Eca109 cells and the xenografted Eca109 solid tumors. The differences in efficacy between HPPH and Photofrin are almost certainly due to the differences in their molar extinction coefficients (~3,000 M/cm for Photofrin at 630 nm; and ~45,000 M/cm for HPPH at 665 nm).

Lethal toxicity induced by various PSs has been documented as early as 1911 (28), and systemic toxicity has been reported following whole body and abdominal light exposure of porphyrin PDT in mice (29). The mechanism of lethality induced by PDT is consistent with traumatic shock syndrome. Endogenous vasoactive mediators of shock include the prostaglandins, the thromboxanes and histamine (28). In the present study, high lethality rates of 62.5 and 50% were observed in the mice that were treated with 1 mg/kg HPPH and 10 mg/kg Photofrin, respectivelym which were lower than that previously reported in several other animal models, which were treated with 0.5 mg/kg HPPH (28). A decrease in the body weights of the mice in the present study was observed during the experimental period (Tables IV and V), indicating that HPPH possessed lower toxicity than Photofrin at the dose that achieved the same efficacy in the mice bearing the Eca109 subcutaneous tumors. A lower lethality was observed in mice following PDT with 0.15, 0.3 and 0.6 mg/kg HPPH over the course of the treatment. This lethality was likely to be tumor-related rather than drug-related, since the control mice exhibited a similar lethality rate (25%; Table V).

PDT efficacy is accompanied by the presence of edema following laser illumination in clinical use, and treated tissues become edematous, followed by the presence of degenerative and necrotic changes. A previous study revealed that the efficacy of HPPH-mediated PDT was accompanied by the presence of mucosal edema within 24 h of laser illumination in carcinogen-induced tumors of the hamster buccal cheek pouch (25). The present study identified that the severity of the edema in the mice after light illumination was dose-dependent. Half of the mice exhibited severe edema following PDT with 1 mg/kg HPPH or 10 mg/kg Photofrin, while half of the mice in the 0.3 and 0.6 mg/kg HPPH-treated groups exhibited slight edema (Table VI; Fig. 6).

Photofrin-mediated PDT-induced tumor tissue damage has been characterized by cell degeneration, with little sign of vascular damage necrosis, edema or severe hemorrhage (30). The histopathological findings in the present study indicated that the tumor tissues in the PS-treated mice demonstrated varying degrees of necrosis (Fig. 7). In addition, a large body of studies have suggested that vascular damage plays a pivotal role in governing the tumor response to PDT in mouse models (31,32). A pioneering study revealed that HPPH-mediated PDT induced an increase in tumor vascular permeability (33). In the present study, the results of the histopathological examination revealed that HPPH and Photofrin exhibited vascular cytotoxicity on the treated tumors (Fig. 7E and F), indicating that vascular damage induced by HPPH-mediated PDT may be a key factor in controlling tumor growth.

In conclusion, the present study demonstrated that the phototoxicity of HPPH-mediated PDT was higher than Photofrin-mediated PDT at the same concentration (dose) *in vivo* and *in vitro*, and that HPPH possessed lower toxicity than Photofrin at the dose that achieved the same efficacy. Thereby, HPPH may be a promising agent for the treatment of human ESCC.

## Figures and Tables

**Table I:** Relative TV in mice following HPPH and Photofrin^®^-mediated PDT over the course of the experiment.

		RTV following treatment					
		
Drug	Dosage, mg/kg	Day 1	Day 5	Day 9	Day 13	Day 17	Day 21
NS	/	1.51±0.33	2.09±0.43	2.91±0.56	3.65±0.51	4.47±0.61	5.50±0.64
Vehicle	1.00	1.60±0.51	2.11±0.74	2.99±0.94	4.11±0.91	4.86±1.18	5.92±1.56
HPPH	0.15	1.64±0.48	1.62±0.35^a^^b^	1.97±0.36^a^^b^	2.44±0.60^a^^b^	3.59±1.37^a^^b^	4.41±1.74^a^^b^
	0.30	1.29±0.43	0.49±0.19^a^^c^	0.52±0.25^a^^c^	0.69±0.30^a^^c^	1.24±0.59^a^^b^^c^	1.79±1.00^a^^b^^c^
	0.60	1.60±0.50	0.31±0.14^a^^c^	0.29±0.14^a^^c^	0.23±0.26^a^^c^	0.30±0.37^a^^c^^d^	0.07±0.09^a^^c^^d^
	1.00	1.41±0.38	0.29±0.12^a^^c^	0.24±0.11^a^^c^	0.19±0.11^a^^c^^d^	0.07±0.16^a^^c^^d^	0.08±0.15^a^^c^^d^
Photofrin	10.00	1.32±0.49	0.40±0.11^a^	0.35±0.02^a^	0.31±0.05^a^	0.16±0.15^a^	0.07±0.08^a^

There were eight mice bearing Eca109 tumors at the start of treatment. Data, with the exception of dosage, are presented as the mean ± SD from 4–8 independent samples. Statistically significant differences were calculated by one-way ANOVA using SPSS 11.5 software.

a P<0.05 vs. the HPPH vehicle group;

b P<0.05 vs. the Photofrin group;

c P<0.05 vs the HPPH 0.15 mg/kg group;

d P<0.05 vs. the HPPH 0.3 mg/kg group.

HPPH, 2-(1-hexyloxyethyl)-2-devinyl pyropheophorbide-a; PDT, photodynamic therapy; NS, normal saline; ANOVA, analysis of variance; TV, tumor volume.

**Table II:** TGI following HPPH- and Photofrin^®^-mediated PDT.

Drug	Dosage, mg/kg	TGI, %
NS	/	92.91
Vehicle	1.00	
HPPH	0.15	74.49
	0.30	30.24
	0.60	1.18
	1.00	1.35
Photofrin	10.00	1.18

TGI values were calculated at the end of the experiment, as described previously. T, mean RTV of treated tumors at the experiment end point; C, mean RTV of control group; HPPH, 2-(1-hexyloxyethyl)-2-devinyl pyropheophorbide-a; PDT, photodynamic therapy; NS, normal saline; TGI, tumor growth inhibition.

**Table III:** Tumor weight inhibition following HPPH- and Photofrin^®^-mediated PDT.

Drug	Dosage, mg/kg	Tumor weight inhibition, %
NS	/	−2.70
Vehicle	1.00	
HPPH	0.15	98.65
	0.30	12.16
	0.60	63.51
	1.00	97.30
Photofrin	10.00	98.65

Tumor weight inhibition values were calculated at the end of the experiment, as described previously. HPPH, 2-(1-hexyloxyethyl)-2-devinyl pyropheophorbide-a; PDT, photodynamic therapy; NS, normal saline.

**Table IV:** Effect of HPPH and Photofrin^®^-mediated PDT on body weights of mice.

		Body weight following treatment					
		
Drug	Dosage, mg/kg	Day 1	Day 5	Day 9	Day 13	Day 17	Day 21
NS	/	21.38±0.97	21.53±1.36	21.93±0.58	22.21±0.57	22.30±0.48	22.46±0.46
Vehicle	1.00	21.38±1.17	21.59±1.29	21.64±1.44	22.22±1.19	22.38±1.05	22.52±1.15
HPPH	0.15	20.88±0.81	19.03±1.22^a^	18.27±0.75^a^	17.90±0.63^a^	17.50±0.58^a^	16.65±1.09^a^
	0.30	20.76±1.16	20.86±1.20	20.78±1.15	20.45±1.22^a^	19.85±1.19^a^	19.37±1.09^a^
	0.60	21.01±0.51	20.69±0.73	20.15±0.86^a^	19.54±0.92^a^	18.99±0.81^a^	18.62±0.79^a^
	1.00	21.18±0.48	20.91±0.62	20.33±0.68^a^	20.14±0.98^a^	18.78±0.61^a^	18.30±0.50^a^
Photofrin	10.00	20.08±1.02	18.91±1.06^a^	18.10±0.56^a^	17.60±0.47^a^	17.34±0.89^a^	16.83±0.17^a^

Body weight was recorded every four days following the treatment. There were eight mice bearing Eca109 tumor at the start of the treatment. Data, with the exception of dosage, are presented as the mean ± SD from 4–8 independent samples. Statistically significant differences were calculated by Student’s t-test using SPSS 11.5 software.

a P<0.05 vs. the HPPH vehicle group.

HPPH, 2-(1-hexyloxyethyl)-2-devinyl pyropheophorbide-a; PDT, photodynamic therapy; NS, normal saline.

**Table V:** Percentage of lethality induced by HPPH- and Photofrin^®^-mediated PDT.

Drug	Dosage, mg/kg	Lethality, %
NS	/	12.5
Vehicle	1.00	25.0
HPPH	0.15	37.5
	0.30	37.5
	0.60	37.5
	1.00	62.5
Photofrin	10.00	50.0

HPPH, 2-(1-hexyloxyethyl)-2-devinyl pyropheophorbide-a; PDT, photodynamic therapy; NS, normal saline.

**Table VI:** Edema arounded the tumors at day 1 post-PDT.

Drug	Dosage, mg/kg	No. of mice	Edema, %			

			None	Slight	Moderate	Severe
NS	/	8	100.0			
vehicle	1.00	8	100.0			
HPPH	0.15	8	100.0			
	0.3	8	25.0	50.0	25.0	
	0.6	8	12.5	50.0	25.0	12.5
	1.00	8		25.0	25.0	50.0
Photofrin^®^	10.00	8		12.5	37.5	50.0

HPPH, 2-(1-hexyloxyethyl)-2-devinyl pyropheophorbide-a; PDT, photodynamic therapy; NS, normal saline.
